# A maize landrace that emits defense volatiles in response to herbivore eggs possesses a strongly inducible terpene synthase gene

**DOI:** 10.1002/ece3.2893

**Published:** 2017-03-21

**Authors:** Amanuel Tamiru, Toby J. A. Bruce, Annett Richter, Christine M. Woodcock, Charles A. O. Midega, Jörg Degenhardt, Segenet Kelemu, John A. Pickett, Zeyaur R. Khan

**Affiliations:** ^1^ International Centre of Insect Physiology and Ecology (ICIPE)NairobiKenya; ^2^ Department of Biological Chemistry and Crop ProtectionRothamsted ResearchHarpendenUK; ^3^ Institute of PharmacyMartin Luther University HalleHalle (Saale)Germany; ^4^ Boyce Thompson InstituteIthacaNYUSA

**Keywords:** (*E*)‐caryophyllene synthase, induced defense, maize landraces, natural enemy, plant–insect interactions, terpene biosynthesis

## Abstract

Maize (*Zea mays*) emits volatile terpenes in response to insect feeding and egg deposition to defend itself against harmful pests. However, maize cultivars differ strongly in their ability to produce the defense signal. To further understand the agroecological role and underlying genetic mechanisms for variation in terpene emission among maize cultivars, we studied the production of an important signaling component (*E*)‐caryophyllene in a South American maize landrace Braz1006 possessing stemborer *Chilo partellus* egg inducible defense trait, in comparison with the European maize line Delprim and North American inbred line B73. The (*E)* **‐**caryophyllene production level and transcript abundance of TPS23, terpene synthase responsible for (*E)* **‐**caryophyllene formation, were compared between Braz1006, Delprim, and B73 after mimicked herbivory. Braz1006–TPS23 was heterologously expressed in *E. coli*, and amino acid sequences were determined. Furthermore, electrophysiological and behavioral responses of a key parasitic wasp *Cotesia sesamiae* to *C*. *partellus* egg‐induced Braz1006 volatiles were determined using coupled gas chromatography electroantennography and olfactometer bioassay studies. After elicitor treatment, Braz1006 released eightfold higher (*E)*‐caryophyllene than Delprim, whereas no (*E)*‐caryophyllene was detected in B73. The superior *(E)‐*caryophyllene production by Braz1006 was positively correlated with high transcript levels of TPS23 in the landrace compared to Delprim. TPS23 alleles from Braz1006 showed dissimilarities at different sequence positions with Delprim and B73 and encodes an active enzyme. *Cotesia sesamiae* was attracted to egg‐induced volatiles from Braz1006 and synthetic *(E)‐*caryophyllene. The variation in (*E)*‐caryophyllene emission between Braz1006 and Delprim is positively correlated with induced levels of TPS23 transcripts. The enhanced TPS23 activity and corresponding (*E)*‐caryophyllene production by the maize landrace could be attributed to the differences in amino acid sequence with the other maize lines. This study suggested that the same analogous genes could have contrasting expression patterns in different maize genetic backgrounds. The current findings provide valuable insight not only into genetic mechanisms underlying variation in defense signal production but also the prospect of introgressing the novel defense traits into elite maize varieties for effective and ecologically sound protection of crops against damaging insect pests.

## 1 Introduction

Terpenes are essential not only for the physiology and development of the plants but also for their interaction with the environment (Gershenzon & Dudareva, 2007; Paré & Tumlinson, 1999; Wink, 2003). Plants produce diverse groups of volatile terpenes, such as monoterpenes and sesquiterpenes, in response to herbivore infestation (Dicke, van Loon, & Soler, 2009; Tamiru, Bruce, et al., 2015). These volatile compounds, often referred to as herbivore‐induced plant volatiles (HIPVs), play a key role in both direct and indirect plant defense (Heil, 2008). Directly, the HIPVs deter phytophagous insects (De Moraes, Mescher, & Tumlinson, 2001; Kessler & Baldwin, 2001) and indirectly, they attract natural enemies antagonistic to the herbivores, for example parasitoids and predators (Dicke & van Loon, 2000; Tamiru et al., 2011; Turlings, Tumlinson, & Lewis, 1990). They may also act as signals to warn neighboring plants against future insect attack (Engelberth, Alborn, Schmelz, & Tumlinson, 2004; Ton et al., 2007).

The diversity of terpene production is attributed mainly to a large class of enzymes known as terpene synthases, which catalyze the formation of a variety of terpene products (Ashour, Wink, & Gershenzon, 2010; Degenhardt, Köllner, & Gershenzon, 2009). Terpene synthases also play a crucial role in determining the unique terpene composition of each taxon (Degenhardt, Köllner, et al., 2009). The biosynthetic pathways that lead to the production of terpenes are well documented (Dudareva Klempien, Muhlemann, & Kaplan, 2013). In maize (*Zea mays*), significant inter**‐** and intra**‐**specific variation in quality and quantity of herbivore‐induced terpene production occurs among different varieties and their wild ancestor, teosinte species (Degen Dillmann, Marion‐Pll, & Turlings, 2004; Rasmann et al., 2005). A loss of defense volatile signals in maize varieties during domestication and artificial selection has been reported, although comprehensive information on the genetic basis is scarce (Köllner et al., 2008; Rasmann et al., 2005; Tamiru et al., 2011). Certain maize landraces emit bioactive compounds including (*E*)‐caryophyllene which recruit natural enemies of herbivores, at an early stage of insect infestation (oviposition); however, this trait is generally rare in the commercial varieties (Tamiru, Khan, & Bruce, 2015; Tamiru et al., 2011; Tamiru, Bruce, et al., 2012).

The spotted stemborer, *Chilo partellus*, is one of the most damaging pests of maize and sorghum in sub‐Saharan Africa, causing yield losses of up to 88% (Kfir Overholt, Khan, & Polaszek, 2002). Since its introduction to Africa from Asia early in the twentieth century (Tams, 1932), the pest has spread rapidly to nearly all countries in sub‐Saharan Africa and different agroecological zones within the countries (Tamiru, Getu, & Jembere, 2007). It has proven to be a very efficient colonizer and a devastating pest wherever it occurs (Tamiru, Getu, Jembere, & Bruce, 2012). Effective chemical control has been hampered by the cryptic feeding behavior of the stemborer larvae and nocturnal habits of the adult moths (Kfir et al., 2002). Furthermore, synthetic insecticides are expensive for the majority of smallholder maize farmers and may result in undesirable consequences such as resistance development by the pest, secondary pest outbreaks, widespread environmental and health risks (Williams & Hammitt, 2001). Hence, developing cost‐effective and environmentally friendly alternative control options such as exploiting natural plant defense and biological control would be timely and highly relevant, especially to subsistence smallholder farmers. The indigenous parasitic wasp *Cotesia sesamiae* (Cameron) effectively parasitize mid‐ to late‐instar stemborer larvae and is widely distributed throughout Africa (Jiang, Zhou, Overholt, & Schulthess, 2006).

A potential to improve crop protection against pest attack by exploiting plant‐derived volatiles and biocontrol agents has been demonstrated in many agricultural settings (Bottrell & Barbosa, 1998; Degenhardt, Hiltpold, et al., 2009; Pickett et al., 2006). The volatile sesquiterpene (*E*)‐caryophyllene is an important signaling component involved in plant defense against harmful pests both above and below ground (Rasmann et al., 2005; Tamiru et al., 2011). However, maize cultivars differ considerably in their ability to produce the defense signal. Furthermore, only certain maize cultivars respond at the egg‐laying stage of pest attack (Tamiru et al., 2011; Tamiru, Bruce, et al., 2012). The current study was aimed at elucidating molecular mechanism for a high level (*E*)‐caryophyllene production by a representative maize landrace of South American origin, “Braz1006,” with egg inducible defense trait. The TPS23 gene activity underpinning induced (*E*)‐caryophyllene emission is functionally characterized in “Braz1006” and compared with the standard European maize line, Delprim and the North American B73 inbred line which has lost TPS23 expression (Köllner et al., 2008). Gene expression analyses of TPS23 gene from maize landraces have never been done previously, and here we conducted parallel investigation of (*E*)‐caryophyllene emission and its regulation mechanisms. The volatile profiles and (*E*)‐caryophyllene synthase (TPS23) gene expressions of Braz1006 were analyzed and compared with Delprim and B73. Furthermore, the (*E*)**‐**caryophyllene synthase encoded by *tps23* was sequenced from Braz1006, Delprim, and B73 to examine any variations that might determine different expression levels of the TPS23 alleles. To clearly define starting time of induction and ensure uniformity of treatments between experimental plants, we used a synthetic elicitor, 6‐substituted indanoyl isoleucine conjugate, a structural analog of the jasmonate isoleucine conjugate that triggers production of bioactive compounds on plants similar to insect herbivory (Schüler, Görls, & Boland, 2001). Response of a parasitic wasp *C. sesamiae* to egg‐induced Braz1006 volatiles and the authentic (*E*)‐caryophyllene standard were determined using a four‐arm olfactometer bioassay and electrophysiological analysis. Studies on the genetic basis of defense signal production by plants coupled with the ecology of tritrophic interactions provide valuable insights into developing a novel and effective pest management strategy against destructive crop pests such as *C. partellus*.

## 2 Materials and Methods

### 2.1 Insects and plants

A culture of *Chilo partellus* (Swinhoe) was initiated from field‐collected stemborers and reared on a semi‐synthetic diet containing sorghum leaf powder (Ochieng, Onyango, & Bungu, 1985) at the International Centre of Insect Physiology and Ecology (*icipe*), Thomas Odhiambo Campus (TOC), Mbita, Kenya. Field‐collected parasitic wasps, *Cotesia sesamiae* (Cameron), were reared on stemborer larvae according to the method described by Overholt, Ochieng, Lammers, and Ogedah (1994). Naïve mated female parasitoids (with no prior exposure to host or plant odor) obtained from the fourth to fifth generation were used in the experiment.

Maize (*Zea mays*) seeds of landrace cv. Braz1006 were obtained from the International Maize and Wheat Improvement Centre (CIMMYT). We also used Delprim (Delley, Switzerland), the B73 inbred line (KWS, Einbeck, Germany). For headspace volatile sampling, the experimental plants were grown individually in pots in insect‐proof screen houses at *icipe*‐TOC, Mbita (0°25′S, 34°12′E) under natural conditions and used in the experiments when 4 weeks old (Figure S1a). For elicitor induction with a synthetic analog, the maize plants were grown in a temperature‐ and relative humidity‐controlled growth chamber (Snijders Scientific, Jumo Imago F3000, Netherlands) with a 16‐/8‐h photoperiod and 1 mmol m^−2^ s^−1^ photosynthetically active radiation (Figure S1b). The temperature and relative humidity were set at 22/18°C (day/night cycle) and 65 ± 5%, respectively.

### 2.2 Volatile collection

Volatile compounds from whole maize plants, with and without stemborer eggs, were collected using headspace sampling (Tamiru et al., 2011). Before volatile collection, plants were kept overnight inside oviposition cages (80 × 40 × 40 cm) into which six gravid female stemborer moths were introduced. Volatiles were collected the following day, starting at the last 2 hr of photophase, for 24 hr as described in Tamiru et al. (2011). Charcoal**‐**filtered air was pumped (500 ml/min) through the inlet port, and volatiles were collected on Porapak Q (0.05 g, 60/80 mesh; Supelco) filters inserted into the outlet port, through which air was drawn at 300 ml/min. Volatiles from control plants were collected as described above but without exposure to stemborer moth egg deposition. After headspace sampling, volatiles were eluted with 0.5 ml dichloromethane and stored at **−**20°C freezer prior to use in bioassays and chemical analysis.

An elicitor treatment that mimics herbivory (Schüler et al., 2001) was employed to ensure uniform start time of induction and similarity of treatments among experimental plants compared to exposure to live insects. The third maize leaves from 2‐week‐old seedlings (*c*. 30 cm high, five expanded leaves) were cut off and incubated in 2 ml tap water with 2.3 μmol/L indanoyl isoleucine conjugates solution for 24 hr (Richter et al., 2015) (Figure S1c). The control plant leaves were kept under identical conditions, by placing freshly cut leaves in a similar volume of tap water, but without elicitor solution. Volatile collection from elicitor‐induced and control Braz1006, Delprim, and B73 plants was conducted using solid‐phase microextraction (SPME). The induced and control plant leaves were frozen in liquid nitrogen and pulverized with a mortar and pestle. Then, an aliquot of 75 mg leaf powder was placed in a screw‐capped gas chromatograph glass vial with a septum in the lid. SPME comprising 100 μm polydimethylsiloxane (PDMS) fiber (Supelco) was placed into the headspace of the vial for 45 min at 40°C. The volatile compounds adsorbed onto the SPME fiber were analyzed by gas chromatography–mass spectrometry (GC‐MS).

### 2.3 Olfactometer bioassays

Responses of parasitoids to headspace volatiles from Braz1006 were tested in a Perspex four**‐**arm olfactometer as described in Tamiru et al. (2011). Air was drawn through the four arms toward the center at 260 ml/min. Headspace samples (10 μl aliquots) were applied, using a micropipette (Drummond “microcap,” Drummond Scientific Co., Broomall, PA, USA), to a piece of filter paper (4 × 25 mm) and subsequently placed in an inlet port at the end of each olfactometer arm. Mated female parasitoids, without previous exposure to plants or hosts, were transferred individually into the central chamber of the olfactometer using a custom**‐**made piece of glass tubing. Time spent in each olfactometer arm was recorded with “Olfa” software (F. Nazzi, Udine, Italy) for 12 min. A parasitoid, which remained motionless for 2 uninterrupted minutes, was considered to be inactive and discarded. The olfactometer was rotated 90° every 3 min during the test to avoid directional bias. The experiments were replicated 12 times. A choice test was carried out in such a way that the two opposite arms held the test stimuli (10 μl aliquots of headspace sample from oviposition‐induced and control plants), while the remaining two arms were blank controls. Behavioral response (attraction) of *C. sesamiae* to the odor stimulus occurred when the parasitic wasps spent significantly more time in the treated region than in the control. Moreover, bioassays were carried out to examine responses of *C. sesamiae* to authentic (*E*)‐caryophyllene standard. Here, the test stimulus (10 μg of synthetic compound) was applied in one of the arms, while the remaining three arms were blank controls.

### 2.4 Gas chromatographic (GC) analysis

GC analysis was performed by injecting 2 μl of headspace sample onto a nonpolar (HP**‐**1, 50 m, 0.32 mm internal diameter, 0.52 μm) capillary column using an Agilent 6890 GC equipped with a cold on**‐**column injector and flame ionization detector (FID). The oven temperature was maintained at 30°C for 2 min and then programmed to increase by 5°C/min to 250°C. Quantification was carried out by calculating and comparing peak areas with known amounts of authentic external standards (Bruce, Midega, Birkett, Pickett, & Khan, 2010; Tamiru et al., 2011). Six replicates of control and induced headspace volatile samples were used. The stereochemistry of linalool was determined using an HP5890 GC equipped with a cool on‐column injector and a FID, fitted with a β‐cyclodextrin chiral capillary column (Supelco, 30 m × 0.25 mm internal diameter, 0.25 μm film thickness). The GC oven temperature was maintained at 40°C for 1 min and then raised by 5°C/min to 150°C, where it was held for 30 min. After confirming successful separation of synthetic enantiomers, co‐injections were carried out. Peak enhancement confirmed the presence of the enantiomer in the headspace sample.

### 2.5 Gas chromatography–electroantennography (GC‐EAG) analysis

#### 2.5.1 Electrophysiology

Electroantennogram (EAG) recordings were made using Ag–AgCl glass electrodes filled with saline solution (composition as in Maddrell, 1969, but without glucose). A female *C. sesamiae* was chilled and the head removed. An indifferent electrode was placed within the head capsule, and the distal ends of the antennae were inserted into the tip of the recording electrode using micromanipulators. The signals were passed through a high‐impedance amplifier (UN‐06, Syntech, the Netherlands) and analyzed using a customized software package (Syntech).

#### 2.5.2 GC‐EAG analysis

Coupled GC–electroantennography was carried out as described in Tamiru et al. (2011) using female *C. sesamiae* antennae and attractive headspace samples of Braz1006. In this setup, the effluent from the GC capillary column was directed simultaneously to the FID detector and antennal preparation. The effluent from GC column was delivered to the antennal preparation by means of a heated transfer line inserted in the side hole of a glass tube (positioned 5 mm from the antenna) through which a purified and humidified airstream (1 L/min) passed continuously over the preparation to minimize desiccation. Separation of the volatiles was achieved on GC (Agilent Technologies, 6890N) equipped with a cold on**‐**column injector and FID. The HP‐1 column comprised a 50 m × 0.32 mm internal diameter and 0.52 μm film thickness. The oven temperature was maintained at 30°C for 2 min and then increased by 15°C/min to 250°C. The carrier gas was helium. The outputs from the EAG amplifier and the FID were monitored concurrently and analyzed using the Syntech software package. The GC peaks were assumed to be active if they elicited responses on three or more of five coupled runs.

### 2.6 Gas chromatography–mass spectrometry (GC‐MS) analysis

Aliquots of attractive headspace samples were analyzed by capillary gas chromatography (GC‐2010, Shimadzu, Duisburg, Germany) directly coupled to a mass spectrometer (GCMS‐QP 2010 Plus, Shimadzu). For analysis of the adsorbed volatiles, the SPME filter was inserted directly into the injector of the GC. The injection temperature was 220°C, and hydrogen was used as a carrier gas with a flow rate of 1 ml/min. An EC5‐MS column (30 m length, 0.25 mm internal diameter, 0.25 μm film thickness) (Grace, Deerfield, IL, USA) was used to separate the volatiles. Ionization was performed by electron impact (70 eV, 250°C). The oven temperature was maintained at 50°C for 3 min and then programmed to increase by 7°C/min to 200°C and 100°C/min to 300°C where it was held for 2 min. The compounds were identified by comparison of retention times and mass spectrometric fragmentation with those of authentic standards and published spectra. Shimadzu software “GCMS Postrun Analysis” was used with the mass spectra libraries “Wiley8” (Hewlett & Packard) and “Adams” (Adams, 2007). GC‐MS identifications were confirmed by peak enhancement with authentic standards on two GC columns of different polarities (nonpolar, HP‐1 column, 50 m, 0.32 mm i.d., 0.52 μm film thickness; polar DB‐wax column, 30 m, 0.32 mm i.d., 0.5 μm film thickness). Six replicates of control and induced headspace volatile samples were analyzed for each line.

### 2.7 RNA extraction and cDNA synthesis

Total RNA was isolated from induced and control maize leaves of Braz1006, Delprim, and B73 with RNeasy Plant Mini Kit (Qiagen Inc., Hilden, Germany) according to the manufacturer's specifications. The isolated RNA was treated with DNaseI, RNase‐free (#EN0521) (Fermentas Inc., St. Leon‐Rot, Germany) to remove residual genomic DNA prior to cDNA synthesis. DNase I‐treated total RNA (3 μl) was reverse transcribed in a 20 μl reaction with RevertAid M‐MuLV Reverse Transcriptase according to the manufacturers’ protocol (Fermentas Inc., St. Leon‐Rot, Germany) using a mix of a random hexamer primer (1 μl) and oligo‐dT primer (1 μl).

### 2.8 Determination of gene transcript levels

Transcript levels were measured by quantitative real‐time polymerase chain reaction (qRT‐PCR). qRT‐PCRs were conducted in 20 μl reactions [10 μl the Brilliant SYBR^®^ Green QPCR Master Mix (Stratagene), 0.5 μl gene‐specific forward primer, 0.5 μl reverse primer, 5 μl cDNA template (1:5 diluted), and 4 μl PCR‐grade water]. Transcript levels were calculated with a standard curve method. The standard curve was generated using a threefold dilution series of cDNA, starting with a 3× concentration of pooled cDNA. A reference gene adenine phosphoribosyltransferase 1 (APT1) was used to control for variation of cDNA amount in each sample. The primer sequences for the reference gene were AGGCGTTCCGTGACACCATC (F) and CTGGCAACTTCTTCGGCTTCC (R). The primer sequences for the gene of interest, *tps23*, were TCTGGATGATGGGAGTCTTCTTTG (F) and GCGTTGCCTTCCTCTGTGG (R). The specificity of all primers was verified by sequencing the respective PCR products.

PCR was performed on a CFX96 Real Time System (Bio‐RAD) with a cycling program consisting of an activation period of the polymerase at 95°C for 10 min, followed by 40 cycles of amplification (95°C for 30 s, 56°C for 30 s and 72°C for 1 min, plate read) and a melting curve from 56 to 95°C. For each reaction, traces of chromatogram outputs and the melting curves were inspected. The ∆‐threshold cycle (∆*C*_T_) for *tps23* gene was calculated relative to the reference gene with the Bio‐Rad CFX Manager program. All reactions were performed in triplicate from the same cDNA template. The *C*_T_ values were derived from an average of three biological samples and three technical replicates each.

### 2.9 Heterologous expression of terpene synthase 23 alleles

The open reading frame (ORF) of TPS23 from maize landrace Braz1006 was amplified with the primer pairs ATGGTAGGTCTCAGCGCATGGCAGCTGATGAGGCAAGATC (F) and ATGGTAGGTCTCATATCAGTCTATTATATCCACATACAATGAATC (R) for expression with a His‐tag (6xHis) at the N‐terminus. The PCR products were cloned with BsaI restriction sites into the bacterial expression vector pASK‐IBA37plus (IBA Biologics GmbH, Göttingen, Germany). The cloning of the TPS23 fragments into the vector yielded a fusion protein with a His‐tag (6xHis) at the N‐terminus. The vector pASK‐IBA37plus without insert was expressed in the empty vector control. The overnight culture of bacteria (*E. coli*, TOP10) harboring the expression constructs was incubated in liquid culture (100 ml ampicillin‐treated LB) at 37°C to OD_600_ (optical density at 600 nm) of 0.6. Expression of the recombinant protein was induced by the addition of 10 μl anhydrotetracycline (1/10 volume, end concentration: 200 μg/L) (IBA Biologics) and incubated at 18°C overnight. Then the culture was centrifuged at 4°C at 4,000 *g* for 10 min to collect the cells, and the supernatant was discarded. The collected cells were disrupted by a 3 × 30 s treatment with an ultrasonicator (Bandelin UW2070, Berlin, Germany) in 3 ml chilled extraction buffer [50 mmol/L Tris–HCl (PH 7.5), with 5 mmol/L sodium ascorbate, 0.5 mmol/L phenylmethylsulfonyl fluoride, 5 mmol/L dithiothreitol (DTT) and 10% (v/v) glycerol]. The cell debris were removed by centrifugation at 11,000 *g* for 20 min, and the supernatant was desalted into 4 ml of assay buffer [10 mmol/L Tris–HCl, (PH 7.5), 1 mmol/L DTT, and 10% (v/v) glycerol] by passage through an Econopac 10DG column (Bio‐Rad). The 6xHis‐tagged enzyme was further purified on a nickel nitrilotriacetate agarose column according to manufacturer's instructions (Qiagen). TPS23 ORF from cDNA of elicitor‐induced leaves of Braz1006, Delprim, and B73 was amplified using the same set of primers shown above and cloned into the pCR4‐TOPO vector for sequencing.

### 2.10 Assay for terpene synthase activity

To test the catalytic activity of the terpene synthase 23 (TPS23) from the maize landrace Braz1006, enzyme assays containing 38 μl of the bacterial extract (TPS23), 50 μl of assay buffer, 10 μl of 10 mmol/L MgCl_2_, and 10µmol/L (E,E)‐FPP mol/L (*E,E*)**‐**FPP were performed in a Teflon**‐**sealed, a screw‐capped gas chromatograph glass vial (1 ml). A SPME mounted with 100 μm PDMS fiber (Supelco) was placed into the headspace of the glass vial for 45 min at 40°C. The adsorbed reaction products were analyzed by injecting the SPME directly on to gas chromatography column (EC05, 30 m length, 0.25 mm internal diameter, 0.25 μm film thickness) coupled to a mass spectrometer (GC‐2010, Shimadzu, Duisburg, Germany). The terpene product was identified by comparison of retention times and mass spectrometric fragmentation with those of authentic standards and published spectra.

### 2.11 Statistical analysis

Parasitoid responses in the four‐arm olfactometer were compared with an ANOVA after converting the data into proportions and log‐ratio transformation, to allow analysis of compositional data. *tps23* expression levels relative to the reference gene (APT1) were analyzed by one‐way ANOVA using general linear model (GLM). Quantification of (*E*)‐caryophyllene was carried out by calculating peak areas with known amounts of authentic external standards (Bruce et al., 2010; Tamiru et al., 2011) and the quantity compared between induced and control Braz1006 plants using a two‐sample T‐test. All data were checked for normality before they were subjected to analysis. Data which lacked normality were transformed using log and square root transformations. Significant means were separated using Student–Newman–Keuls (SNK) test with α set at 0.05. Data analysis was performed using SAS version 9.2 (SAS Institute Inc. 2008).

## 3 Results

### 3.1 Behavioral and electrophysiological responses of parasitoids

**Figure 1:**
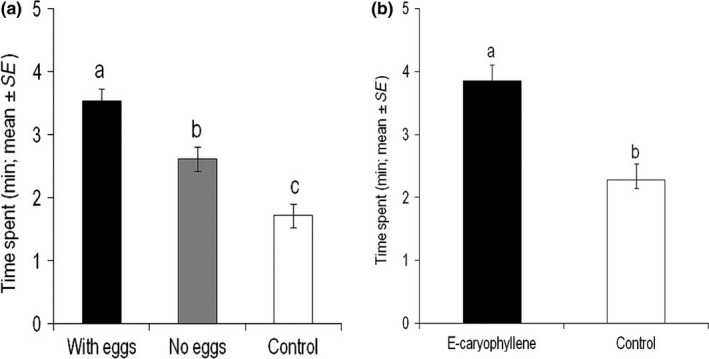
Behavioral responses of the parasitic wasp *Cotesia sesamiae* in a four‐arm olfactometer bioassay to (a) headspace volatiles from maize (*Zea mays*) landrace Braz1006 (Brazil) with and without spotted stemborer (*Chilo partellus*) eggs, (b) authentic standard of (*E*)‐caryophyllene. Each parasitoid was observed for 12 min (*N *=* *12). Mean (± SE) for time spent (min) in each part of the olfactometer is shown. Parasitoid responses were compared by ANOVA after conversion of the data into proportions and log**‐**ratio transformation. Different letters above the bars indicate statistically significant differences based on the Student**–**Newman**–**Keuls (SNK) test (*p *<* *.05)

**Figure 2:**
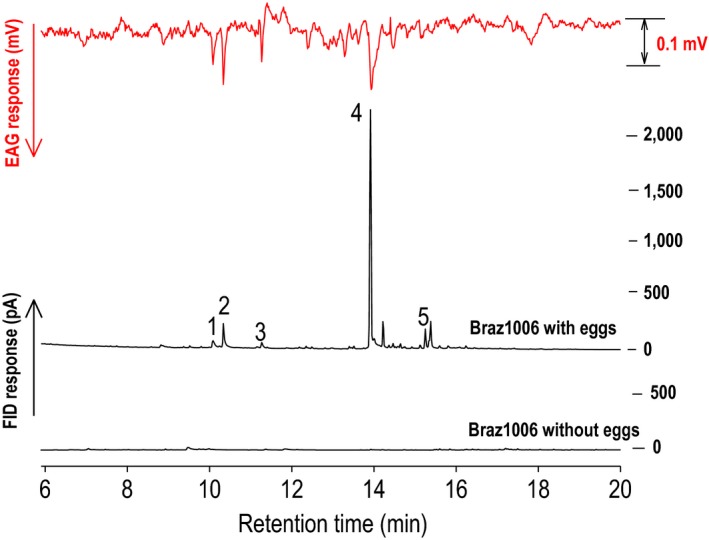
A coupled gas chromatography–electroantennogram (GC **‐** EAG) recording of a female parasitic wasp, *Cotesia sesamiae,* showing responses to different compounds in the headspace volatiles from maize (*Zea mays*) landrace cv. Braz1006 (Brazil) exposed to egg deposition by the spotted stemborer (*Chilo partellus*). The upper trace represents EAG responses of the *C. sesamiae* antenna, whereas the lower traces represent the gas chromatography–flame ionization detector (GC–FID) responses of the headspace volatile sample from maize landrace with and without *C. partellus* eggs. Identified compounds elicited consistent responses from three or more antennae: (1) (*R*)**‐**linalool, (2) (*E*)**‐**4,8‐dimethyl**‐**1,3,7**‐**nonatriene (DMNT), (3) decanal, (4) (*E*)‐caryophyllene, (5) (*E,E*)**‐**4,8,12**‐**trimethyl**‐**1,3,7,11‐tridecatetraene (TMTT).

Female parasitic wasps, *C. sesamiae,* were significantly attracted to HIPVs from maize landrace cv. Braz1006 exposed to stemborer, *C. partellus,* egg deposition (*F *=* *20.87; *p *<* *.0001), compared with volatiles from plants without eggs and blank controls (Figure 1a). GC‐EAG recordings with attractive headspace samples from the landrace revealed that *C. sesamiae* was responsive to several HIPVs, including (*E*)‐caryophyllene and (*E*)‐4,8‐dimethyl‐1,3,7, nonatriene (DMNT) (Figure 2). We chose (*E*)‐caryophyllene to allow comparison across the maize lines tested. Behavioral studies confirmed that (*E*)‐caryophyllene is attractive to *C. sesamiae* (*F *=* *18; *p *=* *.0002) at a 10 μg dose (Figure 1b).

### 3.2 Volatile analysis

**Figure 3:**
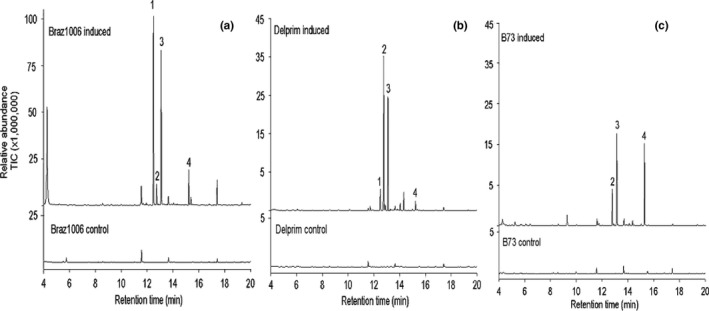
Coupled gas chromatography mass spectrometry (GC‐MS) analysis of volatiles from induced and control maize (*Zea mays*) plants (a) maize landrace Braz1006 (Brazil), (b) maize line Delprim, (c) maize line B73. (*E*)‐caryophyllene (peak 1) was released in large amount (395 ng/g of tissue powder) from Braz1006 compared to Delprim (47 ng/g of tissue powder) (*N *=* *6; *p *<* *.05) after elicitor induction, while B73 did not produce. Other represented EAG‐active compounds were α‐bergamotene (peak 2), (*E*)‐β‐farnesene (peak 3), and (*E,E*)‐4,8,12‐trimethyl‐1,3,7,11‐tridecatetraene (TMTT) (peak 4). Control plants produced only trace amounts of these compounds. Six replicates of control and induced plant volatile samples were analyzed for each maize line

The sesquiterpene (*E*)**‐**caryophyllene was one of the main EAG‐active volatile compounds released by the maize landrace Braz1006 after herbivore egg deposition and elicitor treatment (Figures 2, 3 and S2). A significantly higher quantity of (*E*)‐caryophyllene was released (395 ng/g of tissue powder) by Braz1006 compared to Delprim (47 ng/g of tissue powder) (*t* **‐**test, *p* < .05) (Figure 3a,b), whereas none was detected from the maize inbred line B73 (Figure 3c). Control plants from all maize lines (Braz1006, Delprim and B73) did not release detectable levels of (*E*)‐caryophyllene (Figure 3a–c). Other strongly induced EAG‐active compounds included (*R*)‐linalool, DMNT, decanal, α‐bergamotene, (*E*)‐β‐farnesene, and TMTT (Figures 2, 3 and S2). The mass spectra data of the major bioactive compounds identified inside the headspace samples are shown (Figures S4–S10).

### 3.3 Terpene synthase 23 is strongly induced in the maize landrace

**Figure 4:**
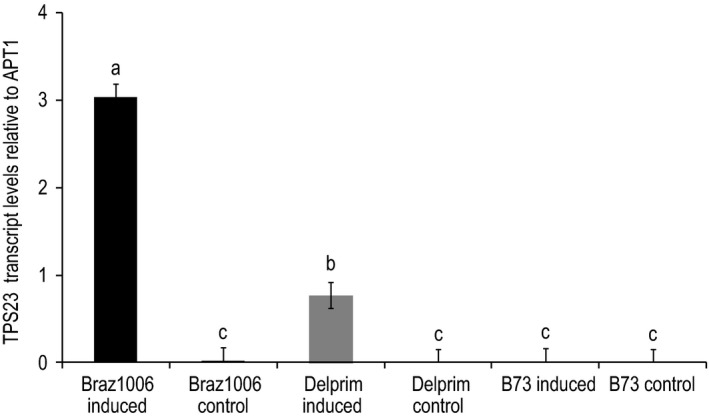
Transcript abundance of *tps23* on control and induced leaves from maize (*Zea mays*) landrace Braz1006 and maize lines Delprim and B73. Transcript levels were determined from 14**‐**day**‐**old plant leaves treated with the elicitor (indanoyl isoleucine conjugates) solution for 24 hr. The *tps23* gene expression was compared relative to the reference gene (APT1). Significance was calculated by one‐way ANOVA from three technical replicates of three biological samples using general linear model (GLM). Different letters above the bars indicate statistically significant differences based on Student–Newman–Keuls (SNK) test (*p *<* *.05)

Relative expression levels determined by qPCR showed significant differences in transcript abundance of *tps23* among the maize lines tested (Figure 4). There was significantly higher accumulation of *tps23* transcripts in the maize landrace after mimicked herbivory compared to Delprim and the controls (*F *=* *5.07, *p *=* *.0099) (Figure 4). On the other hand, there was no significant difference in *tps23* transcript levels between induced B73 and its control (*F *=* *16.42, *p *=* *.9519) where only trace amounts of *tps23* transcripts were detected in either case (Figure 4). The expression levels of *tps23* in Braz1006 and Delprim were positively correlated with the elicitor‐induced levels of (*E*)‐caryophyllene emission in the respective maize lines (Figure 3a,b). On the other hand, the absence of *tps23* transcription in B73 (Figure 4) resulted in a lack of (*E*)‐caryophyllene signal production (Figure 3c).

### 3.4 The TPS23 allele of Braz1006 encodes a (*E*)‐caryophyllene synthase

**Figure 5:**
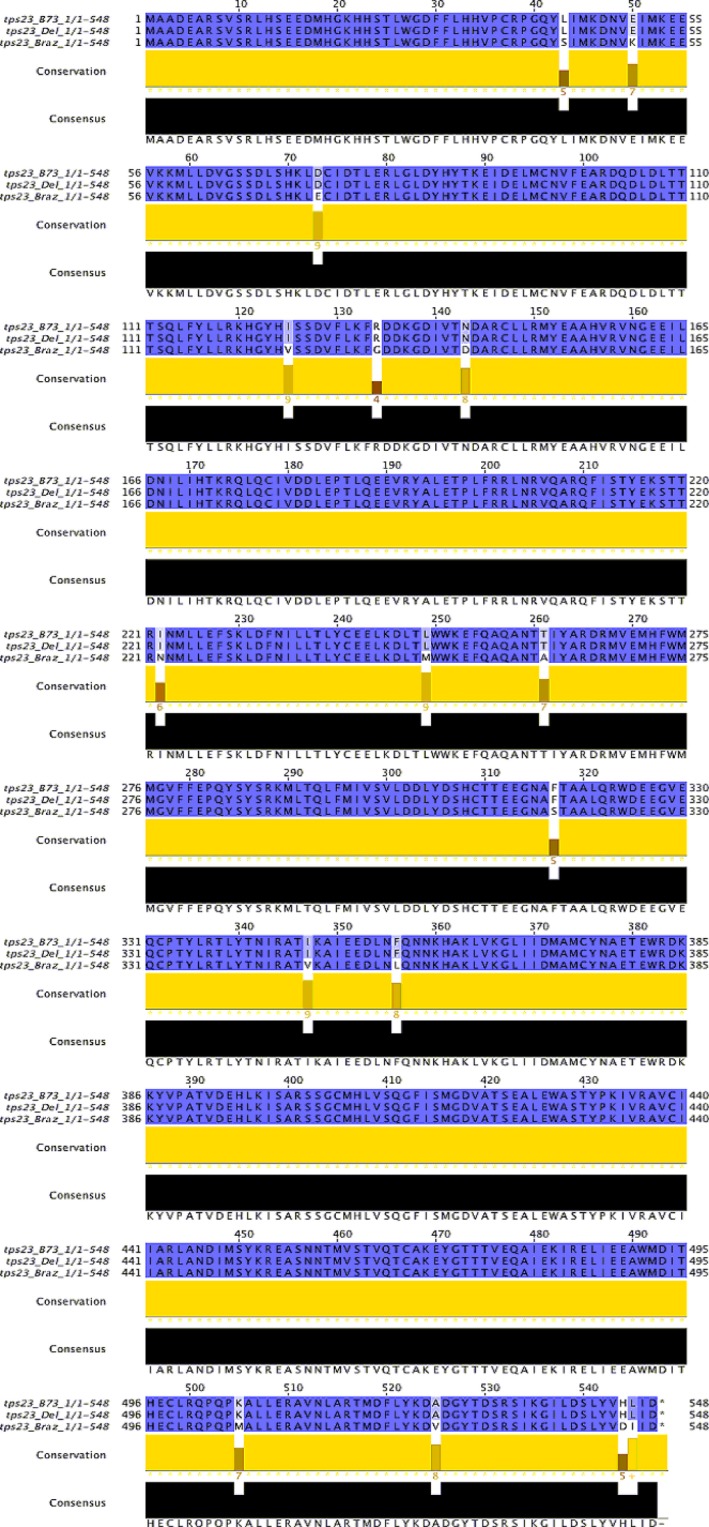
Alignment of the amino acid sequences of TPS23 from the maize (*Zea mays*) landrace Braz1006 and maize lines Delprim and B73. The differences in the amino acid sequences resulting from different TPS23**‐**allels are highlighted in gray

**Figure 6:**
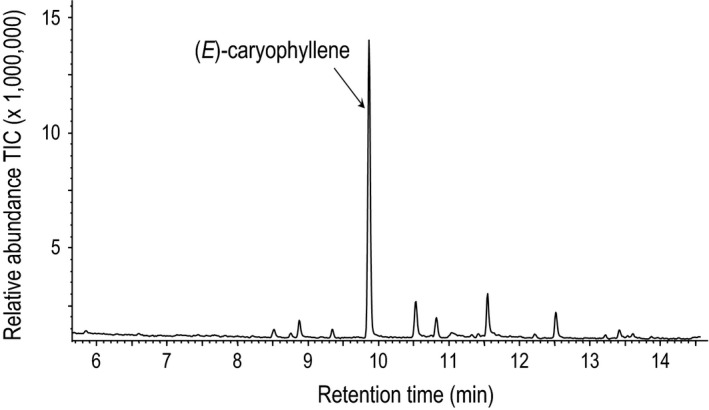
Products of the terpene synthase TPS23. The enzyme was expressed in *Escherichia coli*, extracted, purified, and incubated with the substrate (*E,E*)**‐** FPP. The resulting terpene products were collected with a solid‐phase microextraction (SPME) fiber and analyzed by gas chromatography–mass spectrometry. The major product (*E*)‐caryophyllene was identified by comparison of retention times and mass spectra with those of authentic standards

To analyze variation that might determine different expression levels of the TPS23 alleles, the amino acid sequences from Braz1006, Delprim, and B73 TPS23**‐**alleles were compared. Differences in the gene and amino acid sequences of the alleles were observed between Braz1006 and Delprim (Figures 5 and S3). To ensure TPS23 from the maize landrace Braz1006 is an active (*E*)‐caryophyllene synthase, it was expressed in a bacterial system. Incubation of the recombinant protein with substrate farnesyl diphosphate (FPP; C_15_) resulted in a major product (*E*)‐caryophyllene (Figure 6), which confirmed that the TPS23 allele of the landrace encodes for (*E*)‐caryophyllene synthase. The empty vector control did not show any activity.

## 4 Discussion

Our study provides the first molecular evidence on a novel defense trait expression by a maize landrace in response to both insect egg deposition and herbivory. TPS23, the enzyme catalyzing the final step in the biosynthetic pathway of (*E*)‐caryophyllene, was strongly induced in the maize landrace Braz1006 in response to both *Chilo partellus* egg deposition and an elicitor treatment that mimics herbivory, indicating that the gene encoding TPS23 was active and its expression was inducible. The TPS23 transcript levels were significantly higher, with corresponding superior (*E*)‐caryophyllene production, in the South American maize landrace compared to the European hybrid maize Delprim, after exposure to the elicitor. This was in stark contrast to the North American B73 maize line, which had no such induction of TPS23. The observed differences in amino acid sequences of TPS23 genes of the landrace might have caused these large differences in (*E*)‐caryophyllene production. We are currently developing mapping populations from crosses between these lines to identify SNP (single nucleotide polymorphism) molecular markers that are significantly associated with the trait from segregating populations through genotyping by sequencing and subsequent genome‐wide association study (GWAS). Qualitative and quantitative variations in herbivore‐induced terpene production among maize cultivars have been shown; however, information on the underpinning mechanism like the one reported here is scarce (Degen et al., 2004; Tamiru et al., 2011).

The current study provides further evidence that there is variation among maize varieties in their responsiveness to herbivore attack and implicated regulation of the defense signal biosynthesis in this process. While all three maize varieties contained a potentially active TPS23 allele, the expression levels differed strongly after herbivore induction. This is an intriguing finding and suggests that the same analogous genes have contrasting expression patterns in different maize genetic backgrounds. There was an eightfold higher induction of (*E*)**‐**caryophyllene emission in the maize landrace Braz1006 compared to Delprim, despite the fact that both maize plants were subjected to same concentration and duration of elicitor treatment. The variation in *(E)‐*caryophyllene emission by the maize landrace and Delprim was positively correlated with induced levels of TPS23 transcripts. Recently, Richter et al. (2015) showed that a single farnesyl diphosphate synthase (FPPS3) is induced by herbivory to produce FPP, a substrate required for the formation of the defense volatile sesquiterpenes in maize out of the three functional FPPS. The same study demonstrated similarities in expression kinetics between FPPS3 and the terpene synthase TPS23, implying both genes are regulated by the same signal transduction pathway which is initiated with herbivore induction (Richter et al., 2015). Our study suggests that the signal transduction pathway induces TPS23 much more strongly in the maize landrace Braz1006 than in Delprim, a long‐standing model for emitting herbivore‐inducible volatile signals. This is the first study that gives molecular data on terpene induction of locally adapted maize landrace responsive to early herbivory alert (insect egg deposition).

In contrast to the maize landrace, the defense signal was not produced by maize inbred line B73 after mimicked herbivory. Although adequate evidence on the genetic basis is lacking, an increasing number of studies reported loss of defense traits in plants subjected to artificial selection and breeding (Degenhardt et al., 2009; Palmgren et al., 2014; Rasmann et al., 2005; Tamiru et al., 2011).There was no obvious difference in TPS23 genes sequences between B73 and Delprim. Furthermore, induction pattern of FPPS3, which provides the precursor FPP for the production of the volatile terpene, has been shown to be similar for *(E)‐*caryophyllene‐producing Delprim and B73 (Richter et al., 2015). Hence, the inability to produce (*E*)‐caryophyllene by B73 cannot be attributed to either mutations on the gene itself or lack of FPPS3 activity but may have been caused by inactivation of a key transcription factor or corruption of an enhancer element as suggested earlier by Köllner et al. (2008). In other plant species, for example *Withania somnifera*, upregulation of FPPS activity after herbivory plays a crucial role in controlling terpene‐based defense production (Gupta et al., 2011).

The sesquiterpene (*E*)‐caryophyllene examined in the current study has been shown to elicit electrophysiological and behavioral responses from a key parasitic wasp *C. sesamiae*, widely distributed natural enemy of maize stemborer pest in Africa. The strong induction of defense signals by the maize landrace in response to egg deposition demonstrates finely tuned defense responses sensitive to the earliest stage of herbivore attack. Moreover, emission of oviposition‐induced plant volatiles which attract pest's natural enemies at egg**‐**laying stage of insect attack could provide timely biological control of crop pests since the defense is activated before hatching larvae cause much damage (Fatouros, Cusumano, Danchin, & Colazza, 2016; Tamiru, Khan, et al., 2015). Several strategies have been pursued to increase crop resistance against pest attack by exploiting plant‐derived volatile terpenes (Dudareva & Pichersky, 2008; Khan, Midega, Bruce, Hooper, & Pickett, 2010). For example, the African molasses grass (*Melinis minutiflora)* which emits bioactive compounds such as DMNT and (*E*)‐caryophyllene, attractive to *C. sesamiae* has been used to develop a “push–pull” cropping system for stemborer control by small‐scale cereal farmers in Africa who do not use pesticides (Cook, Khan, & Pickett, 2007; Khan et al., 2010). Emission of these volatile terpenes, in response to herbivore damage by maize and an intact *M. Minutiflora,* can enhance plant resistance by attracting natural enemies and repelling crop pests (Khan et al., 1997; Rasmann et al., 2005; Tamiru et al., 2012; Turlings et al., 1995).

Plants with the ability to produce higher levels of defense volatiles significantly reduce damage inflicted by harmful pests by recruiting natural enemies of herbivores (Degenhardt, Hiltpold, et al., 2009; Hoballah Köllner, Degenhardt, & Turlings, 2004; Khan et al., 2010). On the other hand, these volatile cues enhance foraging efficiency of natural enemies by enabling them to distinguish between mechanically damaged plants and those infested with host pests eventually improving their ecologically fitness (Tamiru et al., 2011). Experimental evidence has demonstrated that the emission of herbivore**‐**induced volatiles can increase plant fitness, in terms of seed production, as a result of reduced plant damage by parasitized herbivores (Dicke & van Loon, 2000; Schuman, Barthel, & Baldwin, 2012). In maize, the potential of optimizing indirect defense by manipulating volatile emissions has been demonstrated (Degenhardt, Hiltpold, et al., 2009). However, the transgenic approach has not been widely used due to marketing issues, influence of pressure groups and regulatory restrictions (Romeis et al., 2008; Tamiru, Khan, et al., 2015). There is an immense potential to employ classical breeding, perhaps enhanced by marker‐assisted selection. The scope for the latter is improved now as we have shown that strongly inducible defense terpene emission trait, responsive even to early egg**‐**laying stage of herbivore attack, exists in locally adapted maize cultivars.

## Conflict of Interest

The authors do not have any real or potential conflicts of interest.

## Data Accessibility and Ethical Approval

All data used in this manuscript are present in the manuscript and its supporting information. This article does not contain any studies with human participants or animals performed by any of the authors.
