# Supplementary material for: A maize landrace that emits defense volatiles in response to herbivore eggs possesses a strongly inducible terpene synthase gene
Source: Ecol Evol. 2017 Mar 21;7(8):2835–45. doi: 10.1002/ece3.2893 (PMC5395458; doi:10.1002/ece3.2893)
Supplement: Supplementary file 1 [file ECE3-7-2835-s001.doc]

**SUPPLEMENTARY INFORMATION**

# **A maize landrace that emits defence volatiles in response to herbivore eggs possesses a strongly inducible terpene synthase** **gene**

Amanuel Tamiru*, Toby J. A. Bruce, Annett Richter, Christine M. Woodcock, Charles A. O. Midega, Jörg Degenhardt, Segenet Kelemu, John A. Pickett and Zeyaur R. Khan

**Figure S1** Experimental maize (*Zea mays)* seedlings inside insect-proof screen house and a temperature (22/18^o^C day/night cycle) and relative humidity (65±5%) controlled growth chamber (Snijders Scientific, Jumo Imago F3000, Netherlands) (a) maize seedlings grown in inside insect-proof screen house under natural conditions (b) maize plants grown inside growth chamber with a 16/8 h photoperiod and 1 mmol m^-2^ s^-1^ photosynthetically active radiation (c) maize leaves inside 2 ml tap water with 2.3 µM elicitor (indanoyl isoleucine conjugates) solution for induction. The control plant leaves were kept under a similar volume of tap water and condition, but without elicitor solution.

**Figure S2** GC profile of headspace volatiles from a maize landrace Braz1006 with and without *Chilo partellus* eggs. The identity of represented EAG active peaks is as follows: (1) (*R*)-linalool (2) (*E*)-4,8-dimethyl-1,3,7-nonatriene (DMNT) (3) decanal (4) (*E*)-caryophyllene (5) (*E,E*)-4,8,12-trimethyl-1,3,7,11-tridecatetraene (TMTT). The sesquiterpene (*E*)-caryophyllene was emitted in significantly higher quantity on a maize landrace Braz1006 exposed to *C. partellus* egg deposition compared to unexposed control (N=6; P<0.05)*.*

**Figure S3** Comparison of nucleotide sequences of *tps23*-Braz1006 with terpene synthase genes of Delprim and B73


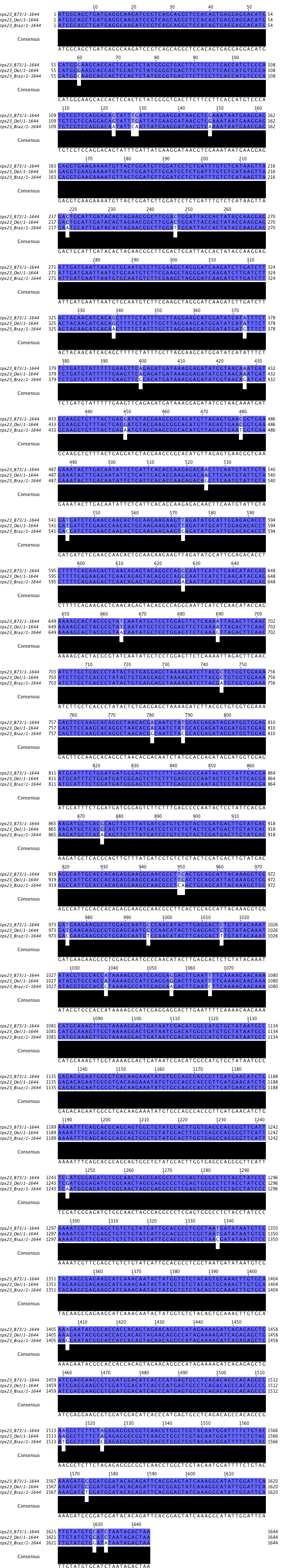


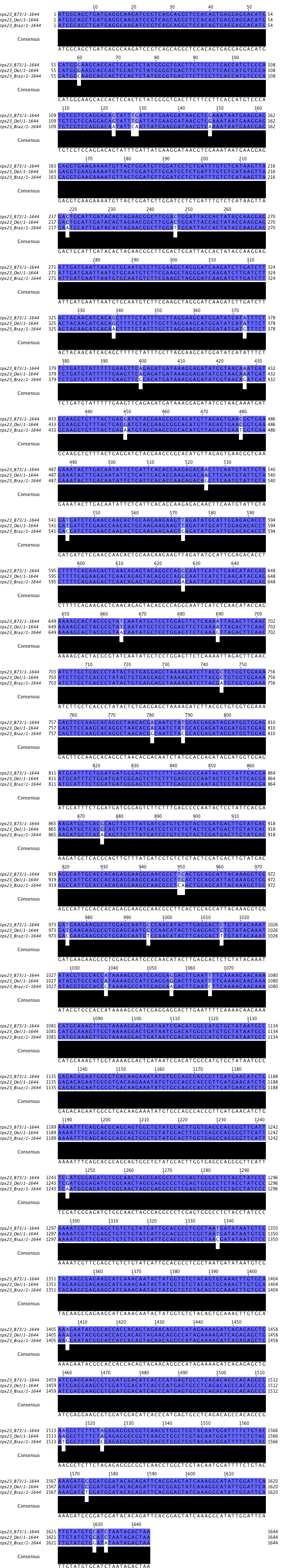


Figure S4 Mass spectra of linalool (2,6-dimethyl-2,7-octadien-6-ol) (a) in the headspace sample (top) and (b) best library match (bottom)

Figure S5 Mass spectra of 4,8-Dimethyl-1,3,7-nonatriene (DMNT) in the headspace sample. Identification of DMNT was possible by comparison of retention times and mass spectrometric fragmentation with those of authentic standards and detecting ion fragments characteristic of the compound as there was no published spectra of the compound in the reference library.

Figure S6 Mass spectra of decanal (Decyl aldehyde, caprinaldehyde) (a) in the headspace sample (top) and (b) best library match (bottom)

Figure S7 Mass spectra of caryophyllene (Bicyclo [7.2.0] undec-4-ene, 4,11,11-trimethyl-8-methylene-, [1R-(1R*,4E,9S*)]-) (a) in the headspace sample (top) and (b) best library match (bottom)

Figure S8 Mass spectra of bergamotene (trans-2,6-Dimethyl-6-(4-methylpent-3-enyl)-bicyclo[3.1.1]hept-2-ene) (a) in the headspace sample (top) and (b) best library match (bottom)

Figure S9 Mass spectra of farnesene (7,11-dimethyl-3-methylene-1,6,10-dodecatriene) (a) in the headspace sample (top) and (b) best library match (bottom)

Figure S10 Mass spectra of (*E,E*)-4,8,-trimethyl-1,3,7,11-tridecatetraene (TMTT) in the headspace sample. Identification of TMTT was possible by comparison of retention times and mass spectrometric fragmentation with those of authentic standards and detecting ion fragments characteristic of the compound as there was no published spectra of the compound in the reference library.
